# The association between diabetes and dermal microvascular dysfunction non-invasively assessed by laser Doppler with local thermal hyperemia: a systematic review with meta-analysis

**DOI:** 10.1186/s12933-016-0487-1

**Published:** 2017-01-19

**Authors:** Dagmar Fuchs, Pepijn P. Dupon, Laura A. Schaap, Richard Draijer

**Affiliations:** 1 Unilever Research and Development, Vlaardingen, Olivier van Noortlaan 120, PO Box 114, 3130 AC Vlaardingen, The Netherlands; 2 Faculty of Earth and Life Sciences, Free University Amsterdam, De Boelelaan 1085, 1081 HV Amsterdam, The Netherlands

**Keywords:** Skin perfusion, Microcirculation, Vasodilation, Morphology, Laser Doppler flowmetry, Laser speckle contrast imaging

## Abstract

**Background/Introduction:**

Diabetes and cardiovascular disease develop in concert with metabolic abnormalities mirroring and causing changes in the vasculature, particularly the microcirculation. The microcirculation can be affected in different parts of the body of which the skin is the most easily accessible tissue.

**Purpose:**

The association between diabetes and dermal microvascular dysfunction has been investigated in observational studies. However, the strength of the association is unknown. Therefore we conducted a systematic review with meta-analysis on the association between diabetes and dermal microvascular dysfunction as assessed by laser Doppler/laser speckle contrast imaging with local thermal hyperaemia as non-invasive indicator of microvascular functionality.

**Methods:**

*PubMed* and *Ovid* were  systematically searched for eligible studies through March 2015. During the first selection, studies were included if they were performed in humans and were related to diabetes or glucose metabolism disorders and to dermal microcirculation. During the second step we selected studies based on the measurement technique, measurement location (arm or leg) and the inclusion of a healthy control group. A random effects model was used with the standardised mean difference as outcome measure. Calculations and imputation of data were done according to the Cochrane Handbook.

**Results:**

Of the 1445 studies found in the first search, thirteen cross-sectional studies were included in the meta-analysis, comprising a total of 857 subjects. Resting blood flow was similar between healthy control subjects and diabetes patients. In contrast, the microvascular response to local skin heating was reduced in diabetic patients compared to healthy control subjects [pooled effect of −0.78 standardised mean difference (95% CI −1.06, −0.51)]. This effect is considered large according to Cohen’s effect size definition. The variability in effect size was high (heterogeneity 69%, p < 0.0001). However, subgroup analysis revealed no difference between the type and duration of diabetes and other health related factors, indicating that diabetes per se causes the microvascular dysfunction.

**Conclusion:**

Our meta-analysis shows that diabetes is associated with a large reduction of dermal microvascular function in diabetic patients. The local thermal hyperaemia methodology may become a valuable non-invasive tool for diagnosis and assessing progress of diabetes-related microvascular complications, but standardisation of the technique and quality of study conduct is urgently required.

**Electronic supplementary material:**

The online version of this article (doi:10.1186/s12933-016-0487-1) contains supplementary material, which is available to authorized users.

## Background

Diabetes contributes to and accelerates cardiovascular disease [1, 2] and macrovascular complications, such as atherosclerosis [3], coronary artery disease [4, 5] and peripheral artery disease [6]. However, the most frequently diagnosed complications in diabetic patients are related to the microcirculation, including foot ulcers [7], retinopathy [8], neuropathy [9], diabetic dermopathy [10] and diminished wound healing [11]. These diabetes-related microvascular dysfunctions can eventually lead to more severe complications, illustrating the necessity to detect microvascular dysfunction in an early stage and identify diabetic patients at risk.

Diabetes may affect the microcirculation in different parts of the body from kidney to eyes and skin, but its function is most easily accessible in the latter. Moreover, skin microvascular function may already be affected in an early stage of the disease as has been assessed by invasive methodology [12].

Microvascular dysfunction used to be determined in feet and toes, as complications are first seen in these body parts. The microcirculation in feet and toes (similar to hands and fingers) can, however, fluctuate substantially due to the presence of arteriovenous anastomoses [13, 14]. Tissue perfusion at these skin sites may therefore not be the most sensitive indicators of the severity and progress of the disease. The forearm and lower leg show a more stable microcirculation and measurements are less invasive. Therefore, the functionality of dermal microcirculation is nowadays more often measured in the forearm and lower leg.

The most commonly used methods to measure microcirculation of the skin are laser Doppler flowmetry (LDF) [15] and laser speckle contrast imaging (LSCI) [16, 17]. These techniques measure the microvascular perfusion, are non-invasive, and provide a continuous measurement [17–20]. Surprisingly, resting dermal microvascular perfusion is apparently not affected in Diabetes, irrespective of the progress of the disease [21, 22]. The functionality of the microvessels may be a more sensitive indicator of complications, and different stimuli are used to determine microvascular response/reactivity [23]. Frequently iontophoresis is used in conjunction with LDF or LSCI. Although iontophoresis is generally considered as being safe, mild local allergic reactions and skin irritations have been observed in some subjects [24]. Another limitation of iontophoresis is that the drug delivery is influenced by skin resistance and this varies considerably between subjects and across different skin areas due to low-resistance pathways in the skin such as sweat ducts or hair follicles [24]. In addition, pH, ion competition in the buffer solution and biological factors such as age, gender, skin hydration and temperature can affect the drug delivery by iontophoresis and thereby contribute to the variation of the measured response. Moreover the iontophoresis procedure is rather time consuming and needs to be repeated at regular intervals [25]. An alternative is local skin heating, which is a stimulus that can be applied to induce local thermal hyperaemia (LTH) due to vasodilation. As LTH is non-invasive, has a good reproducibility and allows to asses different mechanisms causing vasodilation, LTH is one of the most commonly used patient-friendly reactivity tests [20, 23, 26]. Already in the early 90s, changes in dermal blood flow caused by diabetes have been measured with LDF in response to local heating [27]. In the “Methods” section both techniques are described in more details.

The association between diabetes mellitus and microvascular dysfunction as measured by LDF/LSCI with LTH has been investigated in observational studies. However, the strength of the association is unknown. Therefore the aim of this study was to review the strength of the association between diabetes and dermal microvascular dysfunction assessed by LDF/LSCI with LTH. Furthermore the difference in dermal microvascular function between T1DM patients and T2DM patients was assessed.

## Methods

### Selected measurement technique for dermal blood flow

LDF and LSCI are based on the same principle: laser light is directed towards target tissue, usually the skin. Light is scattered back on red blood cells and is then collected and analysed by optical probes [18]. The outcome is presented as blood flow or flux in arbitrary perfusion units, which are proportional to the microvascular perfusion but do not represent actual perfusion values. Nevertheless, these methods have a high sensitivity for the detection of relative changes in blood flow, are well validated, and are particularly used to determine change in perfusion induced by a stimulus at different sites of the skin [16–18, 20, 28, 29]. As shortly described in the introduction, LTH is caused by vasodilation due to local skin heating. The rise in local skin perfusion due to the vasodilation is directly proportional to the skin temperature and reaches its maximum when a local skin temperature of 44 °C is kept for at least 20 min and up to 50 min [23, 30]. The LTH response shows two perfusion peaks which are mediated by independent mechanisms: (1) the initial peak during the first 10 min depends predominantly on local sensory nerves and is mediated by an axon reflex relying on calcitonin-gene-related peptide and substance P, (2) the plateau reached after 20–30 min of heating is primarily mediated by nitric oxide [20, 30–32].

Iontophoresis is a technique in which vasoactive substances are transdermally applied. The underlying principle is the transfer of charged vasoactive drugs using a low-intensity electric current. Several drugs can be used for iontophoresis such as bradykinin, methacholine, and substance P. However, most frequently used drugs are acetylcholine (ACh) and sodium nitroprusside (SNP), which generate an endothelium-dependent and -independent vasodilatation, respectively [23].

In this review, we have focussed on the LTH-skin response in diabetic patients, representing a relatively popular technique, and offering a patient-friendly alternative to most other procedures to assess microvascular dysfunction. Iontophoresis will be discussed to put the result of the meta-analysis into perspective.

### Search strategy

The search strategy was developed and designed by PPD, DF and RD. A systematic search of the databases PubMed and Ovid was conducted including studies up to the 12th March 2015. All possible terms for skin, microcirculation, and glucose metabolism disorders were used as search terms in order to identify all possibly relevant studies. Search terms that were related to the exposure were: skin, dermal, dermis, cutaneous, nailfold, microcirc*, endothel*, microvasc*, microvascular function, iontophoresis, acetylcholine, Ach, sodium nitroprusside, SNP, L-NMMA, local thermal hyper*, heat*, blood flow, perfusion, capillary, vasodilation, laser Doppler, videocapillaroscopy, laser speckle, and Doppler. Search terms in relation to diabetes mellitus were: diabetes, diabetic, DM, insulin and glucose, insulin resistan*, pre-diabet*, HOMA, HbA_1c_, insulin sensitiv*, hyperglyc*, OGTT, oral glucose tolerance test, glucose challenge, and glucose load. Additionally, the search string contained the terms: human, adults, adolescents, subjects, participants, and volunteers. The search was filtered on human studies, articles written in English, and search terms mentioned in the title or abstract.

### Study selection

**Fig. 1:**
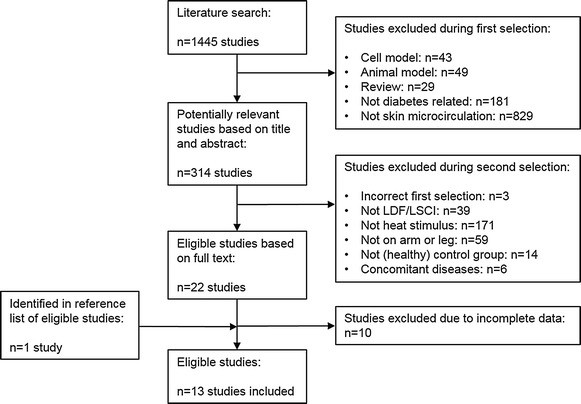
Flowchart of study selection

A two-step approach was followed to identify eligible studies. Both observational- and experimental-studies were deemed eligible, whereas only baseline results were used from experimental studies if included. The flowchart of the selection procedure is depicted in Fig. 1. First, two reviewers (PPD and RD) independently screened title and abstract of all retrieved studies (n = 1445) to identify potentially eligible studies. In a second step, full-texts of the studies were scrutinized by PPD and DF independently to judge eligibility based on the following selection criteria: (1) Laser Doppler flowmeter or LSCI were used to measure microcirculation of the skin, (2) LTH was used as stimulus, (3) measurements were done on arm or leg (not hands, feet, fingers, or toes), (4) studies had both a diabetes group and a (healthy) matched control group, (5) studies were not conducted in patients with any relevant concomitant disease (e.g. no heart failure, dialysis patients). When inconclusive, eligibility was discussed (PPD, RD and DF) until consensus was reached. When relevant results (e.g. mean values, duration of heating) were missing or incomplete, the authors of these studies were contacted to obtain the missing information. If authors did not respond, the study was excluded from the meta-analysis.

### Data extraction and meta-analysis

Baseline characteristics and population details were extracted from all included studies: type of diabetes, methodology to determine diabetes, population size, gender distribution in both diabetes—and control group, age distribution, and BMI. Secondly, information regarding methodology was extracted to identify differences in studies: type of study, specific type of laser Doppler used, duration and temperature of heat stimulus, and location(s) of measurement.

Results were presented in different outcome units by the included studies. Blood flow was presented in arbitrary perfusion units (PU), flux, milliliter × minute^−1^ × 100 g of tissue^−1^, and Volts (1PU is a pre-defined electrical signal in mV). As the blood flow was presented in different units, the results were calculated into standardised mean differences [33]:

$$Standardised \,mean \,difference = \frac{{{\text{Increase in diabetes group}} - {\text{Increase in control group}}}}{Pooled \,standard \,deviation}$$

Mean values and standard deviations of basal flow and peak flow were used to calculate the standardised mean difference for the response to LTH as measured with laser Doppler/LSCI between diabetic patients and healthy control subjects [34]. The SMD is an outcome measure that is difficult to interpret as it does not give an absolute difference or ratio. Cohen designed an effect size index to interpret the importance of the found effect [35]. According to Cohen, an effect size of 0.2 SMD is considered small, 0.5 SMD medium, and 0.8 SMD large.

The meta-analysis was performed in Review Manager 5.3. The *I* ^*2*^ was determined to check for heterogeneity between studies [36]. If the *I* ^*2*^ was 50% or higher, it was assumed there was substantial heterogeneity [37]. If heterogeneity was assumed, subgroup analysis was performed. A random effects model was used in case heterogeneity was not explained by the subgroup analysis.

When the standard deviations of the absolute changes from baseline were not available from individual studies, the missing standard deviations were imputed as described in detail in the Cochrane Handbook [38–40]. The correlation coefficients were calculated from an unpublished validation study performed by Unilever R&D Vlaardingen presenting standard deviations for basal flow (77.6 AU), peak flow (peak 1: 210.1 AU; plateau: 238.4 AU), and change (peak 1 vs baseline: 148.2 AU; plateau vs baseline: 201.0 AU). By using these imputed correlation coefficients, the standard deviations of the change from basal flow to peak flow were calculated for the studies missing the standard deviations.

Several studies contained multiple diabetes groups, e.g. a non-neuropathic diabetes group and a neuropathic diabetes group. These groups were analysed as if they were separate studies. The shared control group, the healthy control subjects, was evenly divided among the two diabetes groups and used as two separate control groups as described in the Cochrane Handbook [38]. The outcome is continuous, therefore the mean change was the same in both control groups.

A priori variables were determined that could have an effect on the outcome. These variables were used to define subgroup analyses. Regarding participant characteristics age (young to old), type of diabetes (type 1 vs type 2), BMI (normal weight to obesity), duration of diabetes (short duration to long duration), and glycated haemoglobin (low HbA_1c_ value to high HbA_1c_ value) were determined as possible influential variables. Regarding methodology the duration of the heat stimulus (<20 min vs >20 min) and location of measurement (forearm vs tibia vs quadriceps) were deemed to be of possible influence.

Eligible studies were also evaluated on quality of conduct and/or reporting. For the quality assessment we used a list of quality criteria that was developed by Downs and Black to assess the quality of observational studies [41]. The criteria were adapted for use for our analysis, involving only studies without medical intervention (Additional file 1). The highest possible quality score was 17 points. The quality scores are presented in Table 1.

**Table 1:** Participant characteristics of studies included in meta-analysis on LTH

First author and year; Ref. no	Group type	N	DM type (N)	Gender M/F	Age (years)	BMI	Duration of DM (years)	HbA1c (%)	BP (mmHg)	Quality score****
Petrofsky et al. [45]	Diabetes	10	NA	NA	65	30.9	NA	7.9 ± 2.1	NA	8
	Control	10	–	NA	55	30.3	–	–	NA	
Aellen et al. [42]	Diabetes + hypertension	1	2	13/8	61	31.5	NA	6.7 ± 0.8	132 ± 16/81 ± 10***	12
	Normotensive control	21	–	10/11	53	23.3	–	–	120 ± 10/78 ± 7***	
Brugler et al. [49]	(a) Diabetic dermopathy	25	1	19/6	51	NA	28 ± 15	7.5 ± 1.5	NA	7.5
	(b) Diabetic control	58	1	27/31	41	NA	23 ± 15.2	8.1 ± 1.5	NA	
	Control	67	–	47/20	47	NA	–	–	NA	
Petrofsky et al. [44]	Diabetes	10	NA	NA	61	33.9	2 to 13	7.4 ± 1.3	NA	6
	Control	10	–	NA	60	27.1	–	–	NA	
Matheus et al. [53]	Diabetes	57	1	24/33	33*	23.7	13* (8–20)**	9.5* (7.5–10.8)**	11 HT	13
	Control	53	–	24/29	27*	23.8	–	5.3* (5.0–5.6)**	0 HT	
Gomes et al. [52]	Diabetes	50	1	21/29	33	NA	15 ± 9.19	NA	NA	9
	Control	46	–	22/24	Matched ± 5 years	Matched	–	NA	NA	
Ngo et al. [51]	(a) Diabetic dermopathy	18	1 (7), 2 (11)	4/14	57	NA	17 ± 12.7	8.4 ± 1.7	NA	6.5
	(b) Diabetic control	24	1 (21), 2 (3)	3/22	42	NA	23 ± 9.8	8.1 ± 2.0	NA	
	Control	48	–	4/44	44	NA	–	–	NA	
Forst et al. [46]	Diabetes	13	1 (6), 2 (7)	7/6	46	NA	4.8 ± 4.9	6.5 ± 1.1	NA	8
	Control	7	–	5/2	38	NA	–	–	NA	
Wigington et al. [50]	Diabetes	61	1 and 2	52/9	58	NA	15 ± 7.8	8.3 ± 1.6	NA	4.5
	Control	41	–	30/11	53	NA	–	–	NA	
Škrha et al. [47]	(a) Microangiopathic diabetes	24	1	12/12	44	24.2	26 ± 9	7.9 ± 1.3	126 ± 14/81 ± 7	10
	(b) Non-microangiopathic diabetes	20	1	9/10	32	23.1	12 ± 8	7.6 ± 1.1	120 ± 12/78 ± 8	
	Control	25	–	12/13	39	23.6	–	4.7 ± 0.3	122 ± 6/77 ± 6	
Arora et al. [54]	(a) Neuropathic diabetes	15	1 (5), 2 (10)	9/6	55	27.2	18.9 (2–35)	NA	NA	9
	(b) Non-neuropathic diabetes	14	1 (4), 2 (10)	8/6	49	27.4	18.3 (1–44)	NA	NA	
	Control	15	–	8/7	48	26.1	–	–	NA	
Morris et al. [43]	Diabetes	14	2	14/0	59	25.9	9.1 ± 7.1	6.5 ± 0.7	89 ± 11.2 (MAP)	9
	Control	14	–	14/0	59	26.2	–	4.8 ± 0.4	95 ± 7.5 (MAP)	
Rendell et al. [48]	Diabetes	35	1	18/17	33	NA	14 ± 5.9	11.3 ± 3.5	NA	7
	Control	30	–	14/16	31	NA	–	–	NA	

If publications only presented the SEM, the SD was calculated by multiplying SEM with the square root of the sample size. *NA* means not available in publication, *HT* hypertensive patients, *MAP* mean arterial pressure

* Mean ± SD or median

** Values between brackets represent the range or interquartile range

*** Mean of visit 1 and 2

**** The maximal quality score that could be obtained was 17

## Results

### Meta-analysis

#### Selection process

The systematic search yielded a total of 1445 studies. During the first selection, studies were excluded that did not meet the criteria (Fig. 1), resulting in the selection of 314 studies. After the second selection procedure, 22 studies were selected as eligible for inclusion in the meta-analysis. The reference lists of these 22 studies were searched for eligible studies, which yielded one additional study. Finally, a number of the eligible studies were excluded from the meta-analysis due to crucial missing results or incorrect methods. Another reason of exclusion was that LTH was not the only stimulus and that therefore the effects of LTH could not be separated from other stimuli. This resulted in the inclusion of 13 studies in the meta-analysis.

#### Overview of included studies

All 13 included studies were analytical cross-sectional studies, investigating the association between a risk factor and outcome at a single point in time. These studies contained a total of 857 participants with the number of participants in each study ranging from 20 to 150 (Table 1).

All studies used a laser Doppler flowmeter. Basal flow was standardised to a normal temperature. These temperatures ranged from 30 to 35 °C. The temperature to obtain maximum vasodilation was 44 °C in most studies. Only two studies used a lower temperature, i.e. 43 °C [42] and 42 °C [43]. The included studies differed on several methodological characteristics. Duration of the heating to obtain maximum vasodilation: in five studies the skin was heated for less than 20 min [42, 44–47], while in eight studies heating was applied for 20 min or more [43, 48–54]. There were three locations of measurements: eight studies measured on the forearm [42, 43, 46–48, 52–54], three on the pretibial surface of the leg [49–51], and two on the quadriceps muscle [44, 45].

#### Pooled overall risk estimate

**Fig. 2:**
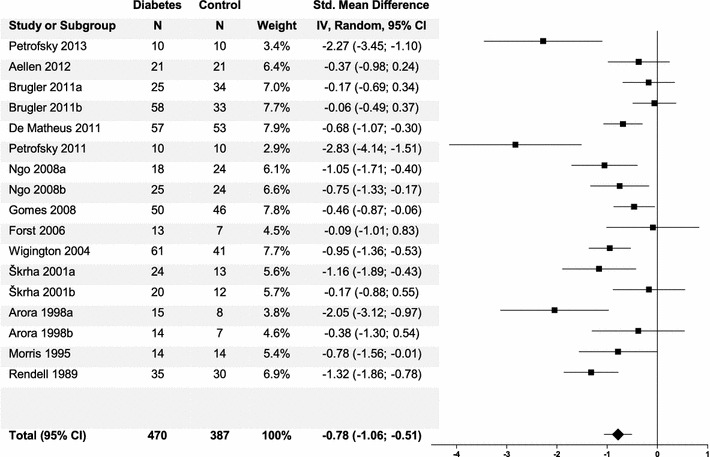
*Forest plot* of pooled overall effect of microvascular function in diabetic patients versus control subjects based on a random effects model. Studies containing subgroups (for instance Arora 1998a and Arora 1998b) were studies presenting two different diabetes groups. The a-subgroups contained diabetic patients with additional complications and the b-subgroups diabetic patients without complications

Baseline dermal blood flow did not differ between control and diabetes group (p = 0.51). The pooled overall estimate showed a lower microvascular response to local thermal stimulus of −0.78 SMD (95% CI −1.06, −0.51) in diabetic patients compared to control subjects (Fig. 2). The mean estimates of all studies showed a lower SMD in the diabetic patients compared to the control group of which eleven subgroups out of ten studies reached significance [43–45, 47, 48, 50–54].

### Heterogeneity analysis

A heterogeneity of 69% (p < 0.0001) was found in the meta-analysis. Therefore heterogeneity was assumed and subgroup analyses were performed. However, the latter could not explain the heterogeneity and a random effects model was used.

### Subgroup analyses

**Fig. 3:**
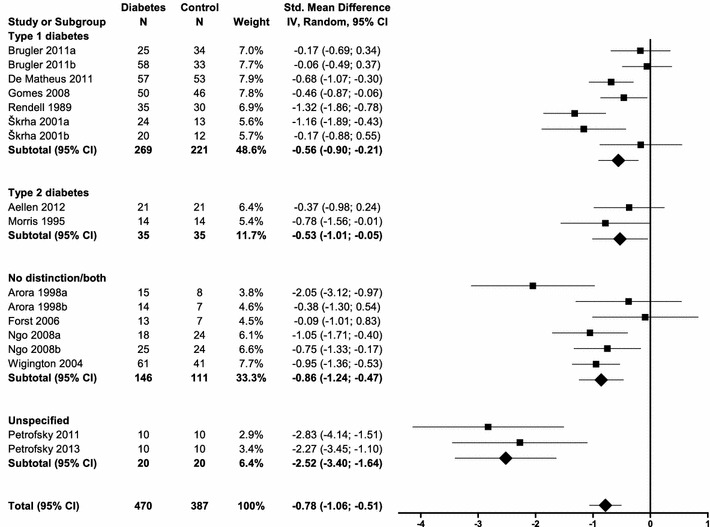
*Forest plot* of subgroup analysis for microvascular function according to type of diabetes

**Fig. 4:**
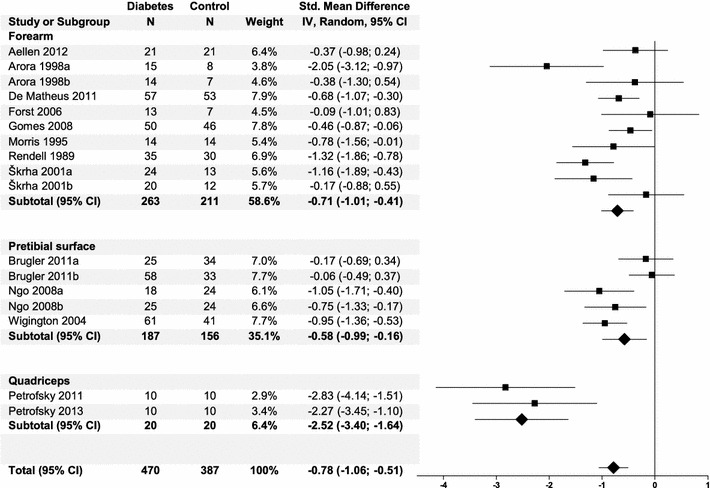
*Forest plot* of subgroup analysis for microvascular function according to location of measurement

A priori, several variables were selected to be of possible influence on the effect. There were no differences between studies conducted on T1DM, T2DM, or both types of diabetes. The studies that did not specify the type of diabetes [44, 45] were significantly different, with an SMD of −2.52 (95% CI −3.40, −1.64, I2 0%) (Fig. 3). However, this difference may also be explained by the unique location of the measurement [44, 45]. Only in these studies blood flow was determined on the quadriceps and showed a significant different effect size compared to studies conducted on the forearm (−0.71 SMD, 95% CI −1.01, −0.41) or pretibial surface (−0.58 SMD, 95% CI −0.99, −0.16) (Fig. 4). Subgroup analyses for the variables age, BMI, duration of diabetes, HbA_1c_, and duration of heating showed no significant effects (data not shown).

Quality of conduct of the eligible studies was moderate (10 studies scored 6–11 out of 17; Table 1).

### Evidence from systematic literature review

#### Iontophoresis

The first selection of studies (n = 314) was checked for studies eligible for a systematic review of the microvascular reactivity after iontophoresis with ACh and SNP. Based on the criteria mentioned in the “Methods” section 22 studies were suitable for inclusion. 6 of the 22 studies did not make a distinction in the types of diabetes [54–59], 6 were conducted in T1DM [52, 53, 60–63] and 11 in T2DM patients [12, 43, 63–70].

Several studies not differentiating between types of diabetes showed a diminished response to ACh [54, 55, 57, 59] and SNP [54, 55, 57] iontophoresis in diabetic patients. However, the differences seem to be more marked and more significant after ACh iontophoresis than after SNP iontophoresis [55, 57]. Studies comparing T1DM patients with healthy controls show similar results. The reduction in blood flow due to diabetes seems to be clearer after iontophoresis with ACh than after iontophoresis with SNP [52, 53, 61]. Next to a reduction in peak vasodilation, Katz et al. [60] showed that time to peak vasodilation after ACh iontophoresis was doubled in diabetic subjects compared to controls, while there was no difference after SNP iontophoresis. Three studies reported a difference in response to both ACh and SNP between patients with T2DM and control subjects [12, 43, 64]. The differences were similar in all three studies and microvascular response was shown to be reduced by a third for both vasoactive substances. A study [63] comparing the microvascular reactivity of patients with T1DM, T2DM, and healthy control subjects, observed a reduced response to ACh in subjects with T2DM, but did not report a difference in response to SNP. In contrast, two studies reported a significant difference between patients with T2DM and control subjects for SNP iontophoresis but not for ACh iontophoresis [68, 69]. The response to SNP iontophoresis was reduced by a third in diabetic patients compared to control subjects. Three studies, however, observed no differences between T2DM patients and healthy control subjects [65–67]. Caballero et al. [12] described a reduced response, assessed as peak vasodilation and percentage increase over baseline, to ACh and SNP in patients with impaired glucose tolerance and relatives of diabetic patients as compared to healthy control subjects. Although not significant, the response gradually decreased from relatives of diabetic patients to subjects with impaired glucose tolerance to diabetic patients [12]. Two of three studies with diabetic patients with microvascular complications (albuminuria, neuropathy or retinopathy) observed at least a trend towards an additional impairment in microvascular function as compared to diabetic patients without complications [54, 69].

## Discussion

The present meta-analysis provides a number of important observations. First, our meta-analyses shows an effect size of the association between diabetes mellitus and microvascular dysfunction as assessed by LTH response that can be considered to be nearly large according to Cohen’s effect size index [35]. Secondly, subgroup analysis showed a difference in effect size between locations of measurement. The fact that the response reported in studies conducted in the quadriceps muscle was more reduced than in the forearm or pretibial surface could suggest that assessment of dermal microvascular function at the quadriceps would be the preferred choice to discriminate healthy from diabetic subjects. However, these findings are limited by the small subgroup size for the quadriceps (n = 40) and being conducted by only one research group [44, 45]. The relatively small standard deviation reported by this group affects the SMD size effect [44, 45] and could either represent the excellence of measurement or a typical smaller variation specifically for the quadriceps as measurement site.

Thirdly, no differences in effect size were found between T1DM and T2DM or whether both types of diabetes were included, which may indicate that the microvascular function is impaired by diabetes per se and not by a factor common to either T1DM or T2DM. Patients with T1DM tend to be younger, leaner, have had diabetes for a longer period and a worse glycemic control compared to T2DM patients [71] and thus could have a poorer microvascular health. However, T2DM patients tend to be overweight and are older in age [71], characteristics that could also impair the microcirculation. Our observation is confirmed by the fact that sensitivity analyses for age, BMI, duration of diabetes, and HbA_1c_ revealed no explanation for the heterogeneity of the data.

Likewise, no differences were found in subgroup analysis for duration of heating. However, the comparison is limited since the data was not reported optimally in a number of studies. The heating procedure should at least take 40 min to allow detection of the nerve-axon reflex-related first peak within the first 10 min, followed by a nitric oxide-dependent plateau-phase at the end of the procedure [72]. In a number of studies the plateau-phase was likely not reached yet. These issues give possible explanations for the lack in differences in subgroup-analyses.

It is interesting to note that four studies included two different diabetes groups: a group of diabetic patients with additional microvascular complications such as dermopathy and a group of matched diabetic patients without these complications [47, 49, 51, 54]. Although not considered a priori, these studies allowed us to differentiate between diabetic patients with and without additional microvascular complications. The findings of these studies indicate that the diabetes group with additional complications had an attenuated response compared to the groups without these complications. Even though the observed difference between these groups was not significant, the attenuated response in diabetic patients with additional complications may support our hypothesis that a longer duration of diabetes and additional complications in diabetic patients are associated with a larger degree of impairment of the microvascular reactivity. In line with our assumption, other groups reported an additional worsening of microvascular function in diabetic patients with microvascular complications [73–75].

As LTH causes vasodilation by different mechanisms, a nerve-axon reflex and a nitric oxide-mediated peak [72], this reactivity test was deemed to successfully assess which aspect is impaired in diabetes. However, due to the lack of guidelines on reactivity tests with a heat stimulus, studies differed in duration of heating. Therefore the difference between the two peaks could not be assessed and the underlying mechanism resulting in microvascular dysfunction in diabetes could not be reviewed. Overall, consensus on standardisation of the microvascular LTH response is required to qualify the method as a patient-friendly diagnostic tool.

The quality of study conduct was moderate. This may reflect either the quality of the study design, but may also be affected by (poor) description and reporting of the study. Therefore, the quality assessment was not used for excluding studies. The quality assessment has revealed that the conduct (or reporting thereof) of dermal microvascular function as assessed by the LTH response in diabetic patients requires improvement. This conclusion supports the recommendation to strive for standardization of the methodology.

Multiple studies reported a reduction in vasodilation in diabetic patients after iontophoresis of particularly ACh [52, 53, 55, 57, 61–63]. This implies that the microvascular dysfunction in diabetic patients is essentially endothelium-dependent. Also, the impairment appears to be more evident in type 1 diabetes than in type 2 diabetes. This may be due to the longer duration of diabetes in type 1 diabetic patients and the worse glycaemic control, which may enhance the microvascular impairment, as an increase in HbA_1c_ values was found to be correlated with a decrease in microvascular response [43, 47, 64, 76, 77]. This correlation is in line with the observation by Caballero et al. [12] that microvascular reactivity gradually decreased from relatives of diabetic patients to subjects with impaired glucose tolerance to diabetic patients. In addition, two studies with diabetic patients with microvascular complications reported at least a trend towards an additional impairment in microvascular function as compared to diabetic patients without complications [54, 69]. However, those effects were not significant and the evidence for a stepwise impairment of microvascular function from subjects at risk for diabetes to diabetic patients with complications is rather limited.

## Strengths and limitations

This review has several strengths. It is the first systematic review on dermal microvascular dysfunction in diabetes and provides a clear insight in this association. Many studies have been conducted addressing this topic, but general consensus was not created yet. Furthermore, we used the PRISMA Statement [78] and the Cochrane Handbook [37] as guidance for this systematic review. This ensures a clear structure of our review and objective methods for data-extraction and -analysis.

Nevertheless, our meta-analysis also has several limitations. Importantly, we observed heterogeneity between studies. Meta-analyses include studies with differences in study design and additionally are prone to be affected by confounding. The number of subjects (n = 857) included was limited, whilst the variance between studies in characteristics of the participants and methodology used may have been too large to reveal the major contributors to the heterogeneity. Laser Doppler and LSCI in combination with local thermal hyperaemia are frequently used methods. Nevertheless, there are no official guidelines available for these measurements and a better standardization is required [73]. Cracowski et al. [20] developed ten suggestions which should be followed to minimize the variability of these measurements. The lack of official guidelines explains why studies differed on methodological aspects such as location of measurement, duration of heating, maximum temperature or outcome measure. The development and implementation of official guidelines for laser Doppler measurements in combination with LTH would improve the reproducibility of the measurement and the comparability of data between studies.

Another limitation was the difference in outcome measures between studies. Not only the unit was different, the absolute values of these outcome measures were also different in range and for this reason we used the SMD. SMD provides an indication of the strength of the association between diabetes and dermal microvascular dysfunction, but cannot be used to determine the absolute difference between diabetic patients and control subjects.

Furthermore, the quality of reporting of the selected studies was not optimal. For example, the gender of the subjects or use of medication is not reported in all publications. This is reflected in the scores the studies received during the quality assessment. Moreover, ten eligible studies were excluded from the analysis due to missing data and no response from the authors, or due to the use of an additional stimulus besides local heating. The excluded studies showed similar results as the included studies and therefore it is unlikely that the exclusion of these studies substantially influenced the findings.

## Conclusions

Diabetic patients show an impaired dermal microvascular hyperaemic response to local heating compared to healthy subjects. An important issue for future research is the implementation of guidelines for microvascular LTH response to strengthen the validity of this tool for assessing (progress of) diabetes-related microvascular complications. Further LDF/LSCI studies including higher number of healthy subjects, prediabetic subjects and diabetic patients with and without complications would be required to demonstrate the stepwise impairment of microvascular function in diabetes and to confirm the applicability of LDF/LSCI as a diagnostic tool.

## Additional file

**Additional file 1.** Quality assessment tool.

## Abbreviations

Ach: acetylcholine
BP: blood pressure
DM: diabetes mellitus
HbA_1c_: glycosylated hemoglobin
LDF: laser Doppler flowmetry
LSCI: laser speckle contrast imaging
LTH: local thermal hyperaemia
SD: standard deviation
SEM: standard error of the mean
SMD: standard mean difference
SNP: sodium nitroprusside
T1DM: type 1 diabetes mellitus
T2DM: type 2 diabetes mellitus
