# Supplementary material for: The association between diabetes and dermal microvascular dysfunction non-invasively assessed by laser Doppler with local thermal hyperemia: a systematic review with meta-analysis
Source: Cardiovasc Diabetol. 2017 Jan 19;16:11. doi: 10.1186/s12933-016-0487-1 (PMC5244618; doi:10.1186/s12933-016-0487-1)
Supplement: Supplementary file 1 — Additional file 1. Quality assessment tool. [file 12933_2016_487_MOESM1_ESM.docx]

Quality assessment tool

|  | **Quality criterion** | **Clarification** | **Scoring** | |  |
| --- | --- | --- | --- | --- | --- |
| *Reporting* | | | | | |
| 1 | Is the hypothesis/aim/objective clearly described? |  | Yes = 1  No/Unclear = 0 |  |  |
| 2 | Are the main outcomes to be measured clearly described in the Introduction or Methods section? | Main outcome = LDF/LSCI with LTH. When mentioned in results = no. When outcomes are defined in detail = yes. When outcomes are mentioned but not defined = partially. | Yes = 1  Partially = 0.5  No/Unclear = 0 | | |
| 3 | Are the characteristics of the subjects included in the study clearly described? | In- and exclusion criteria provided and description of study population characteristics provided. | Yes = 1  No/Unclear = 0 | | |
| 4 | Are the interventions of exposure clearly described? | Exposure = LDF/LSCI with LTH as stimulus. | Yes = 1  No/Unclear = 0 | | |
| 5 | Are the distributions of principal confounders in each group of subjects to be compared clearly described? | List of confounders for microvascular function provided = yes. When only in results indicated = partially. | Yes = 1  Partially = 0.5  No/Unclear = 0 | | |
| 6 | Are the main findings of the study clearly described? | DM status and LDF/LSCI value = yes. | Yes = 1  No/Unclear = 0 | | |
| 7 | Does the study provide estimates of the random variability in the data for the main outcomes? | Reportage of SE, SD, 95%CI, ICR for LDF/LSCI value. | Yes = 1  No/Unclear = 0 | | |
| 8 | Have the actual probability values been reported (e.g. 0.035 rather than <0.05) for the main outcomes except where the probability is less than 0.001? | For LDF/LSCI with LTH as stimulus. | Yes = 1  No/Unclear = 0 | | |
| *External Validity* | | | | | |
| 9 | Were the subjects asked to participate in the study representative of the entire population from which they were recruited? | Entire source population or unselected sample of consecutive patients or random sample = yes. | Yes = 1  No/Unclear/Unable to determine = 0 | | |
| 10 | Were those subjects who were prepared to participate representative of the entire population from which they were recruited? | Same distribution of main confounders in sample and source population = yes. No description of source population = unable to determine. | Yes = 1  No/Unclear/Unable to determine = 0 | | |
| *Internal validity* | | | | | |
| 11 replace | Was an attempt made to blind those measuring the main outcomes of the exposure? | Staff performing or evaluating the LDF/LSCI blinded = yes. | Yes = 1  No/Unclear/Unable to determine = 0 | | |
| 12 | Was an attempt made to blind those measuring the main outcomes of the intervention? | Staff validating the DM status unaware of LDF/LSCI = yes. | Yes = 1  No/Unclear/Unable to determine = 0 | | |
| 13 | If any of the results were based on “data dredging”, was this made clear? | Post-hoc analysis performed without correction for multiple testing = no. If no post-hoc analysis = yes. If no information on post-hoc analysis = unable to determine. | Yes = 1  No/Unclear/Unable to determine = 0 | | |
| 14 | Were the statistical tests used to assess the main outcomes appropriate? | Univariate = no. Multivariate = yes. Weighed nested-case cohort = yes. | Yes = 1  No/Unclear/Unable to determine = 0 | | |
| 15 | Were the main outcome measures used accurate (valid and reliable)? | Determination of LDF/LSCI with LTH reliable = yes. | Yes = 1  No/Unclear/Unable to determine = 0 | | |
| Internal validity - confounding | | | | | |
| 16 | Were the patients in different interventions groups (trials and cohort studies) recruited from the same population? | DM patients and healthy subjects from same hospital/region = yes. Multi-centre with no adjustment = unable to determine. Nothing reported = unable to determine. | Yes = 1  No/Unclear/Unable to determine = 0 | | |
| 17 | Were study subjects in different intervention groups recruited over the same period of time? | When recruitment period (DM patients vs. healthy subjects) not reported = unable to determine. | Yes = 1  No/Unclear/Unable to determine = 0 | | |
| 18 | Was there adequate adjustment for confounding in the analyses from which the main findings were drawn? | Investigation of confounders and consequent adjustment = yes. If “multivariate” is mentioned, but not specified which variables were adjusted for = unclear. If partly adjusted = partially. | Yes = 1  Partially = 0.5  No/Unclear = 0 | | |
| *Power* | | | | | |
| 19 | Was the power sufficient to detect a clinically important effect where the probability value for a difference being due to a chance is less than 5%? | When not described = unable to determine. | Yes = 1  No/Unclear/Unable to determine = 0 | | |
|  | | | | | |
